# Disentangling the Evolutionary Cause–Effect Relationships of Environment, Sexual Selection, and Body Size With Birdsong Frequency

**DOI:** 10.1002/ece3.73351

**Published:** 2026-04-03

**Authors:** Hector Fabio Rivera‐Gutierrez, Osvaldo Alexander Gomez‐Gomez, Valentina Montoya‐Jaramillo, Felipe A. Toro‐Cardona, Paula Pinzon‐Cardenas

**Affiliations:** ^1^ Grupo de Ecología y Evolución de Vertebrados, Instituto de Biología, Facultad de Ciencias Exactas y Naturales Universidad de Antioquia Medellín Colombia; ^2^ Red de Biología Evolutiva Laboratorio de Bioclimatología, Instituto de Ecología, A.C. Carretera Antigua a Coatepec 351, El Haya Veracruz México

**Keywords:** acoustic adaptation, birdsong evolution, morphological constraint, neotropical birds, phylogenetic path analysis, sexual selection

## Abstract

Multiple selection pressures act simultaneously and interactively to shape trait evolution, yet their combined effects are often overlooked. We used Phylogenetic Path Analysis to examine the causal relationships between birdsong frequency, body size, habitat structure, and sexual selection across 472 Neotropical passerine species. This approach quantifies direct and indirect effects while comparing alternative evolutionary scenarios. A single causal model consistently explained variation in song frequency across 100 phylogenies. Tree cover increased minimum, peak, and maximum frequencies but did not affect bandwidth. Sexual dimorphism reduced bandwidth and influenced frequency, whereas morphological traits constrained frequency parameters and differentially shaped bandwidth. Habitat structure and sexual dimorphism also affected morphology, generating additional indirect effects on song frequency, and tree cover further influenced sexual dimorphism. Together, these results reveal an integrated network of environmental, sexual, and morphological drivers of acoustic evolution, supporting hypotheses of sexual selection, acoustic adaptation, and morphological constraints. Our findings highlight that trait evolution emerges from interacting processes, and that simple linear models may fail to capture the complexity of these evolutionary pathways.

## Graphical abstract

We used Phylogenetic Path Analysis to disentangle the causal relationships linking birdsong frequency with body size, environment, and sexual selection. Our results show that these factors interact through both direct and indirect pathways, revealing a complex network of evolutionary influences. This integrated framework highlights that simple linear models are insufficient to capture the multidimensional processes shaping acoustic evolution.

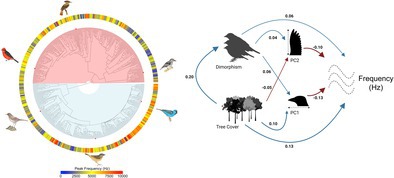

## 1 Introduction

Animals use signals from different sensory modalities (visual, tactile, acoustic, electrical, chemical) to communicate in different contexts (Bradbury and Vehrencamp 2011), with acoustic signals being widespread and predominant in different taxonomic groups, including insects (e.g., cicadas, katydids) and both aquatic (fish) and terrestrial vertebrates (Bradbury and Vehrencamp 2011; Chen and Wiens 2020; Wilkins et al. 2013) (e.g., amphibians, mammals). Acoustic communication encompasses a wide diversity of signals, production mechanisms, and functions involved in communicative interactions that play a fundamental role in different evolutionary aspects, such as fitness, speciation, and adaptation (Bradbury and Vehrencamp 2011; Wilkins et al. 2013). Birds are well known for using acoustic signals in a variety of communicative interactions, including mate attraction, territorial defense, parent‐offspring communication and group cohesion, among others, which play a pivotal role in species fitness, survival and evolution (Bradbury and Vehrencamp 2011; Catchpole and Slater 2008).

Birdsong exhibits remarkable diversity in complexity, temporal structure, and spectral characteristics, generating species‐specific acoustic signatures that often facilitate recognition (Bradbury and Vehrencamp 2011; Odom et al. 2021; Wilkins et al. 2013). Among these traits, frequency is one of the most variable and ecologically relevant components, influencing sound transmission and propagation (Barker 2008; Morton 1975) as well as the information content of the signal (Gil and Gahr 2002; Bradbury and Vehrencamp 2011). Variation in frequency characteristics across species can arise from multiple evolutionary and ecological pressures, including sexual selection, environmental conditions, body size, and phylogenetic history (Bradbury and Vehrencamp 2011; Marler and Slabbekoorn 2004; Slabbekoorn and Smith 2002). Sexual selection, in particular, has long been proposed as a major driver of acoustic divergence through mate preferences and territorial interactions (Catchpole and Slater 2008; Searcy and Anderson 1986), potentially contributing to reproductive isolation and speciation (Price 1998; Seddon and Tobias 2007). Understanding how these ecological and evolutionary factors interact to shape frequency evolution remains a central question in birdsong research (Morton 1975; Ryan and Brenowitz 1985).

Although sexual selection is often assumed to influence song frequency, the underlying mechanisms are difficult to distinguish. Species that rely on acoustic communication and are subject to sexual selection pressures may exhibit low‐frequency songs if selection favors larger male body size, indirectly constraining frequency through biomechanical scaling rules linking body size to sound production (Fletcher 2004; Ryan and Brenowitz 1985). Conversely, sexual selection may favor greater vocal performance or signal complexity—such as broader bandwidth or increased modulation—rather than directional shifts in frequency per se (Searcy and Anderson 1986; Gil and Gahr 2002; Catchpole and Slater 2008). Thus, sexual selection can act both directly on acoustic structure and indirectly via size dimorphism (Andersson and Iwasa 1996; Price 1998), making the predicted association between sexual selection and frequency inherently ambiguous. These intertwined causal pathways highlight the need for analytical approaches capable of modeling complex cause–effect relationships among correlated traits (Gonzalez‐Voyer and von Hardenberg 2014; Hardenberg and Gonzalez‐Voyer 2013).

Although sexual selection is considered a major force shaping birdsong characteristics, recent studies suggest that sexual selection may not play a role in driving frequency differences between species (Friis et al. 2021; Sagar et al. 2024). In addition, the morphological constraint hypothesis (Ryan and Brenowitz 1985) suggests that differences in body size may account for variation in species frequency. Since the relationship between the dimension of a sound‐source and the wavelength produced establishes the efficiency of sound radiation (Bradbury and Vehrencamp 2011; Bennet‐Clark 1998), there is a negative relationship between body size and song frequency (Ryan and Brenowitz 1985; Bradbury and Vehrencamp 2011; Gil and Gahr 2002). In general, it is expected that large species are able to produce lower‐frequency songs (Ryan and Brenowitz 1985; Mikula et al. 2021; Fletcher 2004), in contrast with small species. Finally, song frequency is also related to transmission efficiency and attenuation in different environments (Bradbury and Vehrencamp 2011; Barker 2008; Morton 1975). In general, vegetation structure may cause scattering and absorption of acoustic signals leading to greater attenuation of higher frequencies than lower frequencies (Barker 2008), causing low‐frequency songs to transmit better in forest habitat (Morton 1975). This idea is known as the acoustic adaptation hypothesis (Barker 2008; Rothstein and Fleischer 1987).

Evolution of phenotypic traits is not mediated by a single factor; instead, multiple forces may act concurrently to shape evolutionary trajectories. In fact, a trait can respond to several selection pressures simultaneously, often driving variation in different directions (Gallagher et al. 2024; Dunn et al. 2015). Another possibility is that a trait evolves as a by‐product of the evolution of a different trait (Gould and Lewontin 1979), or through genetic or functional correlations with other traits (Price and Langen 1992). Finally, a trait may also represent an exaptation, having been co‐opted from a structure or feature that originally evolved for another purpose (Winchell et al. 2023). Although this framework also applies to acoustic signals, to date there are relatively few studies that explicitly examine how this complex web of interactions drives the evolution of acoustic communication. For example, song frequency (a key acoustic trait) may simultaneously respond to natural selection mediated by habitat structure or density and to sexual selection via mate choice. Indeed, natural selection has been found to influence song frequency indirectly via its effects on body size (Hay et al. 2024; Derryberry et al. 2018). Moreover, given the prominent role that sexual selection plays in shaping body size (Andersson and Iwasa 1996; Andersson and Simmons 2006), it is not surprising that body size strongly affects birdsong frequency (Hall et al. 2013), potentially mediated by mate preferences. In addition, the strength and direction of sexual selection are not independent of environmental context (Blanckenhorn 2005; Schütz and Taborsky 2005). Habitat structure can influence mating systems, population density, and the costs and benefits of sexually selected traits, thereby constraining or amplifying the evolution of dimorphism. Therefore, it is essential to investigate the evolution of acoustic signals using integrative approaches that consider multiple selection pressures and their interactions.

Hypotheses regarding the evolution of acoustic signals—including sexual selection, morphological constraints, and acoustic adaptation—have been examined extensively, yet few studies have evaluated these factors simultaneously (Mikula et al. 2021; Friis et al. 2021; Sagar et al. 2024). Nevertheless, the statistical approaches used in these studies do not fully disentangle cause–effect relationships among variables because multiple selection pressures and their interactions are not modeled concurrently. Most analyses rely on phylogenetic generalized least‐squares (PGLS) regressions (Mikula et al. 2021; Friis et al. 2021) or taxonomically informed mixed‐effect models (Sagar et al. 2024) that may quantify linear associations among traits while accounting for phylogenetic non‐independence (Garamszegi 2014; Mikula et al. 2021). Given that natural and sexual selection may impose selection pressures on different factors simultaneously and in an intricate manner, PGLS is limited in its ability to uncover causal pathways among factors (Hardenberg and Gonzalez‐Voyer 2013).

To address this limitation, it is important to use a methodological approach that studies all factors and their interactions simultaneously to disentangle evolution. In this study, we aim to understand the evolutionary relationship between birdsong frequency, body size, environment, and sexual selection by modeling all possible interactions within a causal network. To solve this, we implemented a Phylogenetic Path Analysis (PPA) using data from 472 neotropical passerine species (228 Oscines, 244 Suboscines). PPA allows for the estimation of direct and indirect effects and the relative importance of alternative causal models (Gonzalez‐Voyer and von Hardenberg 2014). We therefore propose that PPA provides a more powerful framework to disentangle evolutionary cause–effect relationships underlying variation in birdsong frequency (Hardenberg and Gonzalez‐Voyer 2013; van der Bijl 2018).

## 2 Methods

### 2.1 Acoustic Data

We obtained song recordings from Macaulay Library and Xenocanto databases. Recordings from Macaulay were in WAV format, sampling rate: 44KHz, 16 bit, and recordings from Xenocanto were in MP3 format. Recordings on Xenocanto are rated according to their quality, ranging from A (loud and clear) to E (barely audible). In Xenocanto, recordings per species were organized according to their quality, and only those rated A or B were downloaded. All recordings were inspected before download, and if the quality matched the rating, they were used in the analysis. We visually inspected the recordings and selected songs based on their duration, composition, and structure. We excluded calls (short, simple, repetitive sounds) from our analysis. Xenocanto recordings were transformed using Ocenaudio V.3.6.3 to match the characteristics of Macaulay's recordings. We assumed that MP3 recordings would be suitable because a previous study suggested that, on average, MP3 compression does not generate systematic deviations in acoustic measurements and that repeatability in acoustic variables measured on compressed and uncompressed files is high (Araya‐Salas et al. 2019). All recordings were normalized and filtered using low‐pass (±8–10 KHz)/high‐pass (±1 KHz) filters if needed. Recordings were analyzed using Avisoft (Avisoft SAS‐LAB Pro V.5.2, Berlin, Germany). First, spectrograms of all recordings were visually inspected to determine their quality (signal‐to‐noise ratio). Spectrogram parameters: Hamming window, FFT Length 512, frame size 75%, overlap: 50%. Recordings that were of sufficient quality were considered in our analysis. At least three different recordings per species, and a minimum of five strophes per recording were analyzed. Strophe boundaries were detected automatically in Avisoft using a −30 dB threshold relative to the peak amplitude within each strophe. Because recordings were pre‐filtered and only high signal‐to‐noise files were retained, this threshold excluded background noise while capturing the full temporal extent of each strophe, including lower‐amplitude elements. This approach minimized subjectivity in manual segmentation and avoided truncation of biologically relevant variation. We selected the −30 dB threshold because it provided a consistent criterion that balanced exclusion of background noise with retention of low‐amplitude song elements, ensuring that biologically relevant variation was not truncated. Frequency parameters (minimum, maximum, and peak) were then extracted from the averaged spectrum of each selection; here, the threshold refers to spectral limits and follows the logic described by Brumm et al. (2017). Peak frequency was defined as the frequency at the maximum amplitude in the spectrum, while minimum and maximum frequencies were obtained from the averaged spectrum within the limits set by the threshold used to segment the strophes. Bandwidth was calculated as the difference between the maximum and minimum frequencies.

Because our dataset includes both uncompressed (.wav) and compressed (.mp3) recordings, we considered the potential effects of compression on frequency measurements. Previous work (Araya‐Salas et al. 2019) shows that although mp3‐to‐wav conversion may introduce additional variance, these deviations are not systematic and do not bias frequency estimates directionally. This has led many recent comparative bioacoustics and macroevolutionary studies (Mikula et al. 2021; Friis et al. 2021; Sagar et al. 2024) to rely on high‐quality recordings from online repositories without requiring sensitivity analyses restricted to .wav files. In our dataset, .wav recordings represent a small and taxonomically uneven subset; restricting analyses to this subset would reduce sample size and introduce phylogenetically non‐random missing data. For these reasons, and following current best practices, we included all high‐quality recordings that met our signal‐to‐noise and quality criteria, regardless of compression format.

### 2.2 Morphological and Sexual Selection Data

A total of nine morphological measurements from males (culmen, bill depth, bill width, gape, wing length, tail length, tarsus length, hallux, body mass) were used to evaluate morphological variation. Measurements were obtained from a published dataset for Colombian species (Montoya et al. 2018) by measuring museum specimens following a standardized protocol (Lopez‐Ordoñez et al. 2016) or from a database collected by the Ecology and Evolution of Vertebrates Research Group. Since both the published dataset and the database included several individuals per species, average values were calculated for each species. We visited the Museo Universitario de la Universidad de Antioquia and the Museo de Ciencias Naturales de La Salle in Medellín to collect measurements of museum specimens. OG collected all measurements, and several individuals per species were measured and averaged.

We conducted a Principal Component Analysis (PCA) with Varimax rotation for dimensional reduction in R software. This analysis revealed that the included morphological variables could be summarized into two components, which together explained 86% of the cumulative variability. The first component, which explained 63.29%, was more closely associated with culmen measurements (0.85), bill height (0.90), bill width (0.92), and tarsus length (0.92). The second component accounted for 22.74% of the total variability and was more closely associated with measurements related to body mass (0.21), wing length (0.16), and tail length (0.21). PC scores for each species were used in subsequent analysis (Figure S1). In addition, we used a proxy for sexual selection, namely sexual dimorphism, which was calculated as the difference between the log of the male tarsus length and the log of the female tarsus length of each species. This variable is referred to as “Dimorphism” hereafter. Positive values indicate that males are larger than females, which is related with intensity of sexual selection (Székely et al. 2000). To achieve this, we used the Avonet dataset (Tobias et al. 2022). As there was insufficient data for all species, we used a phylogeny‐based procedure to impute the missing data for 42 species.

### 2.3 Environmental Data

To include the influence of environment on species song frequency evolution, we calculated the mean tree cover percentage within each species' distribution range. For this, we used the species distribution polygons from BirdLife (BirdLife International and World 2023) and the tree canopy cover raster data from Hansen et al. (2013). This raster layer is defined as canopy closure for all vegetation higher than five meters, expressed as a percentage within each 30‐m grid cell. To improve computational efficiency, we processed the tree cover data for the Americas using Google Earth Engine and resampled it from its original 30‐m resolution to a 1‐km resolution, employing the mean value for each resampled cell. Finally, we applied the zonal statistics tool in ArcGIS Pro (ESRI 2024) to estimate the mean tree cover percentage within the distribution range of each species.

### 2.4 Phylogenetic Tree

**FIGURE 1:**
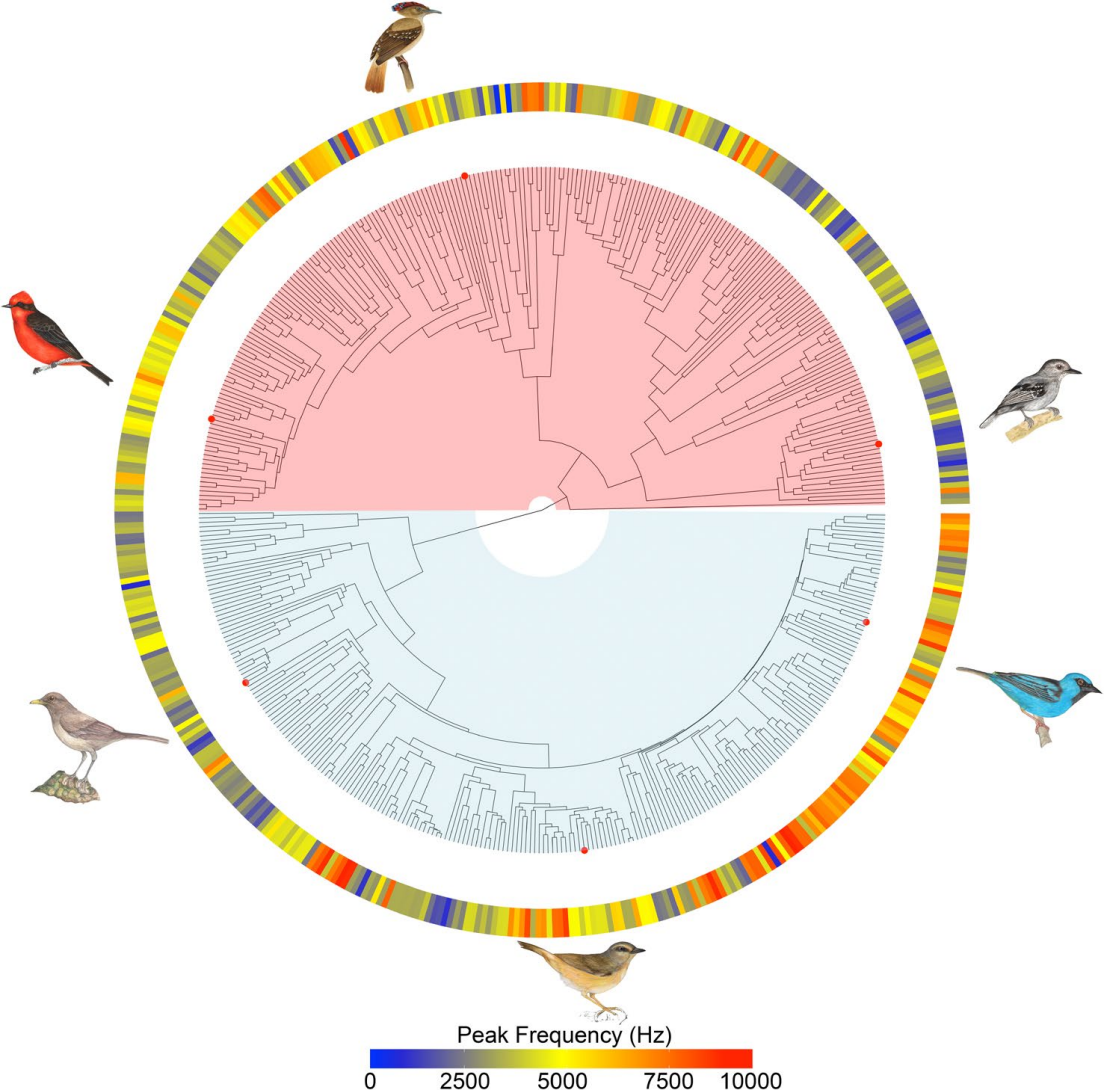
Phylogenetic tree representing variation in Peak frequency. Color of the ring represents frequency (Hz) ranging between 100 and 10,000 Hz. The species are illustrated from top left, clockwise: *Pyrocephalus rubinus, Onychorhynchus coronatus, Thamnophilus atrinucha, Dacnis cayana*, *Myiothlypis fulvicauda* , and *Turdus grayi* .

We selected 472 Passeriform species belonging to different families (228 Oscines, 244 Suboscines). To build a reliable phylogeny, we used a recently published complete bird phylogeny (Mctavish et al. 2025) which provides a standardized phylogeny with a robust, validated background. This is suitable in the absence of a complete phylogenetic analysis of all the species in our study. Implementing a random imputation procedure for 10 species that were not included in the McTavish phylogeny, a total of 100 different trees were generated with the help of the rtrees package in R (Li 2023). All trees were used in the analysis to account for phylogenetic uncertainty. A phylogenetic tree combining the Mctavish et al. (2025) phylogeny with our frequency data was generated using the ggtree and ggtreeExtra packages in R (Yu 2022) to illustrate both species diversity and the trait variation examined in our analyses (Figure 1).

### 2.5 Phylogenetic Path Analysis (PPA)

PPA is a phylogenetic comparative method that builds a set of structural equations to test causal relationships. For each response variable (maximum, minimum, peak frequency, and bandwidth), we built a total of 13 different models, including a null model. The structure of each model represented a unique combination of cause–effect relationships, and each response variable received the same models. We used habitat structure (Tree cover percentage), Dimorphism, and two principal components (PC) from morphology as factors. Given that the environment may have an impact on sexual size dimorphism (Blanckenhorn 2005; Schütz and Taborsky 2005), we explicitly considered the possibility that environmental structure (tree cover) affects sexual dimorphism in our causal framework. Furthermore, morphology was set as a response to Tree cover and Dimorphism to infer the cause–effect relationship. A graphical summary of the built models is presented in Figure S2.

The analysis for each response variable was performed using the 100 different phylogenetic trees. Therefore, a total of 5200 models were run (1300 for each response variable). The most informative model for each response variable was selected based on the corrected information criterion (CICc) for small samples. Models with a delta CICc lower than two (CICc of the model minus the lowest CICc) were considered the most informative (Hardenberg and Gonzalez‐Voyer 2013; van der Bijl 2018). When interpreting model results for each response variable, we recorded how often each of the 13 models had ΔCICc ≤ 2 across the 100 phylogenetic trees. The conditional average coefficient of the most frequent models was calculated to present the results (Hardenberg and Gonzalez‐Voyer 2013). PPA were implemented with the help of the phylopath package in R (Hardenberg and Gonzalez‐Voyer 2013, van der Bijl 2018).

## 3 Results

### 3.1 Model Selection Across Phylogenetic Uncertainty

Model support was highly consistent across the 100 phylogenetic trees for all response variables. Models with ΔCICc ≤ 2 were considered equally informative. Across all frequency parameters, a single causal model was consistently supported, being the only model included within the ΔCICc ≤ 2 confidence set and obtaining ΔCICc = 0 across all trees.

This supported model includes direct effects of environmental structure (Tree cover) and sexual selection (Dimorphism) on frequency parameters, as well as indirect effects mediated by morphology. In addition, environmental structure directly affects sexual dimorphism, indicating that habitat structure plays a role in modulating sexual selection regimes. A complete summary of model support across phylogenetic trees is provided in the Supporting Information.

Because the same model was consistently supported across all trees and response variables, subsequent interpretation focuses exclusively on this causal structure. To account for phylogenetic uncertainty, parameter estimates were averaged across the 100 phylogenetic trees. All phylogenetic trees were derived from a well‐resolved avian backbone phylogeny, with variation introduced through the imputation of a small number of species absent from the original tree. Model selection consistency across trees therefore reflects robustness to this source of phylogenetic uncertainty.

### 3.2 Causal Structure of the Supported Model

**FIGURE 2:**
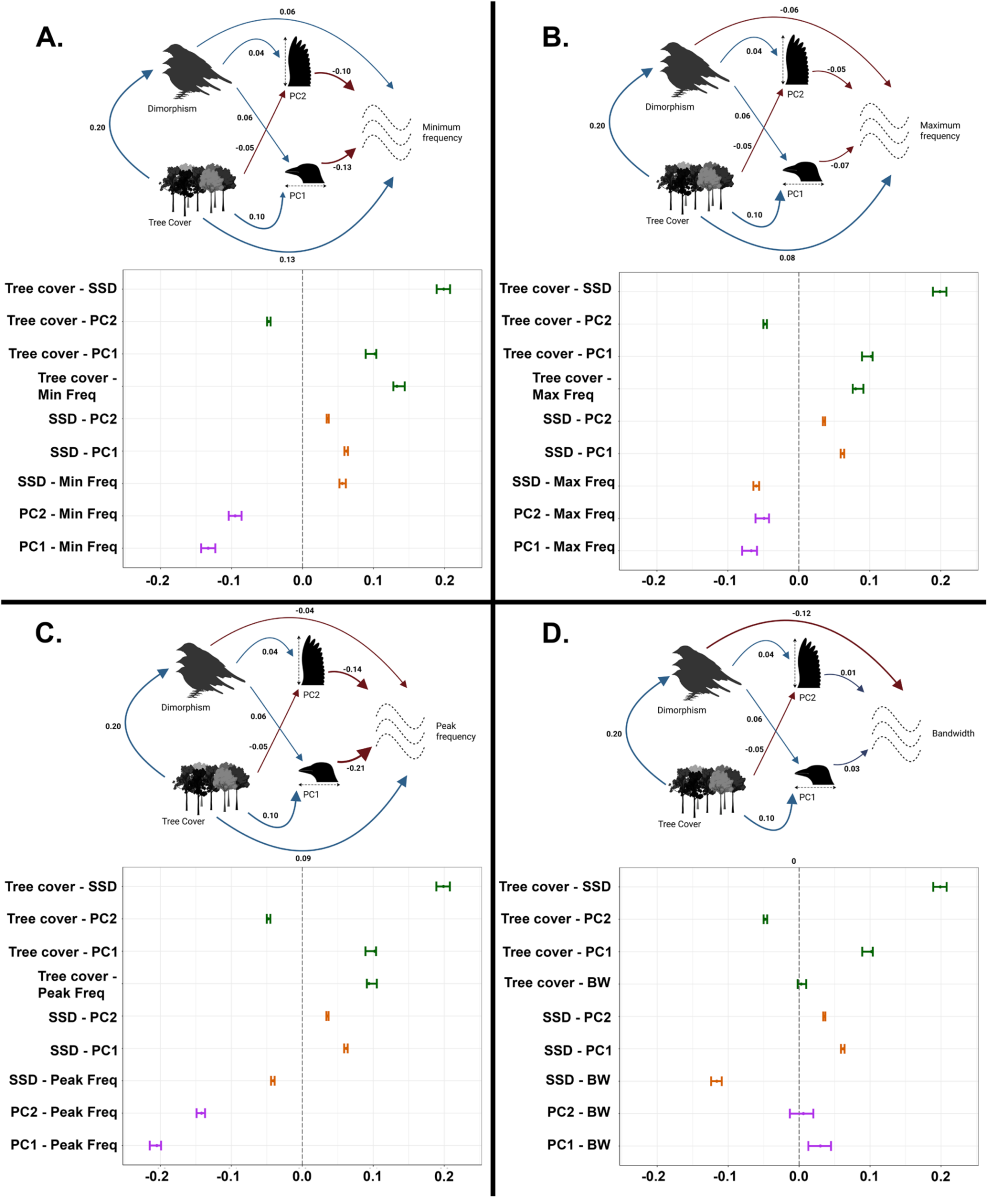
Results for phylogenetic path analysis (PPA) on birdsong frequency. Each panel shows cause–effect relationship between variables on the top, and averaged correlation coefficients between pairs of variables on the lower part. Panels show results for *A. minimum* frequency, B. Maximum frequency, C. Peak frequency and D. Bandwidth. In the models, thickness of the lines represents significance of the effect, numbers indicate the coefficient, and sign refers to the direction of the relationship.

The supported causal model indicates that variation in song frequency is shaped by a combination of direct and indirect effects of environmental structure, sexual selection, and morphology (Figure 2). Tree cover exerted both direct effects on frequency parameters and indirect effects mediated by sexual size dimorphism (SSD) and morphology. In turn, Dimorphism affected frequency both directly and indirectly through its effects on morphological components.

### 3.3 Environmental Effects (Acoustic Adaptation)

Tree cover showed consistent positive direct effects on minimum, peak, and maximum frequency, which contradicts predictions of the acoustic adaptation hypothesis (Figure 2 and Table 1). In contrast, Tree cover showed no clear association with bandwidth, indicating that environmental structure influences absolute frequency parameters but not frequency dispersion.

**TABLE 1:** Averaged standardized coefficients ±S.E. of relationship between factors and responses.

Coefficients						±S.E.			
Response	Factors	SSD	PCA2	PCA1	Response	SSD	PCA2	PCA1	Response
*Minimum frequency*	Tree cover	0.199	−0.048	0.102	0.133	0.051	0.054	0.048	0.040
	SSD		0.035	0.062	0.056		0.049	0.043	0.036
	PC2				−0.095				0.036
	PC1				−0.133				0.054
*Maximum frequency*	Tree cover	0.199	−0.048	0.102	0.080	0.051	0.054	0.048	0.036
	SSD		0.035	0.062	−0.060		0.049	0.043	0.032
	PC2				−0.049				0.048
	PC1				−0.067				0.054
*Peak frequency*	Tree cover	0.199	−0.048	0.102	0.094	0.051	0.054	0.048	0.038
	SSD		0.035	0.062	−0.041		0.049	0.043	0.034
	PC2				−0.142				0.050
	PC1				−0.205				0.057
*Bandwidth*	Tree cover	0.199	−0.048	0.102	0.003	0.051	0.054	0.048	0.036
	SSD		0.035	0.062	−0.116		0.049	0.043	0.033
	PC2				0.006				0.049
	PC1				0.030				0.055

In addition to its direct effects on frequency, Tree cover affected morphology and SSD, revealing indirect pathways through which environmental structure shapes acoustic evolution.

### 3.4 Sexual Selection

Sexual selection, quantified by SSD, had both direct and indirect effects on song characteristics (Figure 2). SSD was directly associated with all frequency parameters, including bandwidth, although the direction and magnitude of these effects differed among parameters. SSD also indirectly influenced frequency through its positive effects on morphology (Table 1). These results indicate that sexual selection contributes to acoustic evolution not only through direct selection on signal structure but also via an indirect effect mediated by body size and morphology.

Notably, SSD had a strong negative effect on bandwidth, suggesting that sexually dimorphic species tend to produce signals with narrower frequency ranges, independent of environmental effects.

### 3.5 Morphological Constraints

Morphology exerted consistently strong effects on acoustic traits across all frequency parameters (Figure 2). Both morphological components were negatively associated with minimum, peak, and maximum frequency, indicating that larger‐bodied species produce lower‐frequency signals. In contrast, bandwidth showed a different pattern: PC1 was positively associated with bandwidth, while PC2 showed no clear effect (Table 1).

These results provide support for the morphological constraint hypothesis and highlight that different aspects of morphology may differentially affect frequency parameters and bandwidth.

### 3.6 Integrated Effects Across Hypotheses

Taken together, these results show that variation in birdsong frequency and bandwidth emerges from the interaction of environmental structure, sexual selection, and morphology. While Tree cover primarily affects absolute frequency parameters, sexual selection and morphology influence both frequency and bandwidth, albeit through distinct pathways (Figure 2, Table 1). This integrated causal framework underscores the importance of simultaneously evaluating multiple evolutionary drivers to understand the evolution of acoustic signals.

## 4 Discussion

We tried to understand the evolutionary relationship between body size, environment, sexual selection, and birdsong frequency by using a PPA that may help to disentangle the evolutionary cause–effect relationship between factors and response variables. Our study suggests that the most complex model is the best‐supported model along 100 different tree topologies, being the most frequent result, suggesting that the evolution of birdsong is regulated in a very complex way, in contrast to previous studies that considered single factors in a linear way. The evolution of birdsong frequency is affected by all factors simultaneously, with different intensities, and the factors also interact with each other.

### 4.1 Environmental Effects (Acoustic Adaptation)

Our analysis reveals both direct and indirect effects of the environment (tree cover) on song frequency. Tree cover had little direct influence on bandwidth but increased minimum, peak, and maximum frequencies. It also had a positive effect on PC1 (beak and tarsus length) and a negative effect on PC2 (body mass, wing length, and tail length). Because morphology negatively affects frequency parameters, these environmentally driven shifts in morphology generate additional indirect effects on song frequency. Environmental variation may impose differential selection pressures on morphological traits through habitat specialization, leading species to occupy distinct regions of morphological space. For example, certain environments may favor maneuverability (short and round wings) (Reif et al. 2016) or resource‐use traits such as larger beaks. Thus, the environment influences frequency both directly and indirectly via its effects on morphology.

Although the direct effect of tree cover contradicts the acoustic adaptation hypothesis, similar patterns—namely weak or absent direct environmental effects on frequency—have been reported in previous studies (Mason and Burns 2015; Derryberry et al. 2018; Mikula et al. 2021; Sagar et al. 2024), This growing body of evidence suggests that habitat structure may not impose strong direct selection on frequency traits. Instead, environmental influences on acoustic evolution may operate primarily through indirect pathways, particularly via their effects on morphology.

The direct effect of environmental structure (tree cover) on sexual size dimorphism suggests that habitat structure modulates sexual selection regimes rather than acting solely as a background variable. Structurally complex habitats may influence encounter rates, signal transmission, and competitive interactions, thereby affecting the strength and direction of sexual selection acting on body size (Seddon et al. 2013; Tobias and Seddon 2009; Shine 1989). Ecological gradients have been shown to shape sexually dimorphic traits through interactions between sexual and natural selection, with habitat‐specific constraints influencing the expression of sexually selected traits (Beltrán et al. 2022). In addition, environmental structure can affect mating systems, territoriality, and population density, all of which are known to modulate sexual selection intensity in birds and, consequently, patterns of sexual size dimorphism at macroevolutionary scales (Barber et al. 2024; Cally et al. 2021).

### 4.2 The Role of Sexual Selection

Our analysis indicates that sexual selection, evaluated through sexual size dimorphism, was positively associated with morphology, a pattern consistent with female preferences for larger body sizes. It is well known that females do not only prefer larger individuals (Andersson and Iwasa 1996), but also larger ornaments (Andersson and Simmons 2006; Andersson and Iwasa 1996; Møller 1988). Evolution of this preference may be explained by different mechanisms, such as direct phenotypic benefit, *Fisherian* evolution, or Indicator mechanisms (Andersson and Simmons 2006). It is clear that individual differences in body size are related to male quality (Searcy et al. 2004), and it may provide advantages during male‐to‐male interactions (Andersson and Iwasa 1996). Therefore, female preference for large body size may provide both fitness and ecological benefits (Andersson and Simmons 2006), causing directional selection that may affect positively the mean value of body size of bird species. Alternatively, the relationship between sexual size dimorphism and morphology may partly arise as a consequence of sex‐specific allometric scaling, whereby divergence in body size between males and females generates correlated differences in morphological traits. Although sexual selection has been identified as an important driver of dimorphism in birds (Dale et al. 2007), allometric relationships may still contribute to the observed patterns independently of direct selection on morphology. Therefore, the effect of sexual dimorphism on morphology should be interpreted as reflecting a combination of sexual selection and allometric constraints. This interpretation is consistent with the causal framework evaluated in our phylogenetic path analysis, which allows sexual selection and allometric effects to act simultaneously through direct and indirect pathways.

### 4.3 Morphological Constraints

As expected, morphology negatively affects frequency, except for bandwidth. Therefore, the effect of morphology on frequency parameters supports the morphological constraint hypothesis (Ryan and Brenowitz 1985). At the same time, sexual selection has a negative effect on Maximum and Peak frequency, suggesting a preference for lower‐frequency songs. This preference may be developed as an indicator signal (Nowicki and Searcy 2004) suggesting that low frequency is a reliable and honest signal of body size in birds. The lack of a relationship between morphology and Bandwidth may be explained by the relationship between body size and frequency parameters. If all frequency parameters vary together, then bandwidth may be similar between species of different sizes and may not change with differences in body size. The complex interaction between sexual selection, morphology, and frequency resulted in direct and indirect selection pressures acting concurrently for low‐frequency songs and providing support for the role of song frequency as an honest signal of body size.

### 4.4 Integrated Effects Across Hypotheses

Recent studies tried to understand the role of body size, sexual selection, habitat and evolutionary history on birdsong frequency at a global scale (Mikula et al. 2021; Sagar et al. 2024). While they did not find support for the acoustic adaptation hypothesis, we provide evidence of an indirect effect of environment on frequency parameters. In addition, they provide contrasting results for the sexual selection hypothesis. The different analytical approaches clearly yield different results. Both PGLS and PPA are widely used methods for understanding evolutionary patterns across multiple species (Garamszegi 2014). However, while a PGLS explains variance due to one or different factors in a linear way, a PPA integrates the PGLS and predicts the set of conditional probabilities if causal effects are true (Hardenberg and Gonzalez‐Voyer 2013; van der Bijl 2018). In addition, PPA integrates these associations into a causal framework, estimating conditional dependencies and quantifying both direct and indirect effects (Gonzalez‐Voyer and von Hardenberg 2014). This makes PPA particularly suited for disentangling the combined influence of natural and sexual selection on birdsong evolution.

Differences between our findings and those from global PGLS analyses may also reflect variation in data sources and sampling scales. Although our dataset is more modest in size, it is internally consistent, with acoustic, morphological, and environmental data collected within a single country and often from the same locations. This reduces potential biases associated with broad geographic sampling, especially when multiple traits are involved. For these reasons, we consider PPA a more appropriate tool for identifying evolutionary cause–effect relationships among the factors shaping birdsong. Future work could provide a more comprehensive assessment by expanding phylogenetic sampling, integrating broader environmental variables, and exploring different dimensions of sexual selection. Such extensions would complement our internally consistent dataset while situating our findings within a wider comparative framework.

As a conclusion, our PPA suggests that both sexual and natural selection play a role in the evolution of birdsong frequency in a direct and indirect way through an effect on morphology, providing evidence for the Morphological constraint, Sexual selection, and Acoustic adaptation hypotheses. Here we have shown that the evolution of birdsong is regulated in a complex way, where both natural and sexual selection play a fundamental role in shaping morphology and driving the evolution of acoustic characteristics. Sexual selection acts in concert with natural selection, influencing body size in birds and affecting song frequency in different directions. Phylogenetic Path Analysis offers a powerful framework for uncovering these causal relationships, revealing that evolutionary processes operate through interconnected pathways that simple linear models cannot fully capture.

## Author Contributions

**Hector Fabio Rivera‐Gutierrez:** conceptualization (lead), formal analysis (equal), investigation (equal), methodology (equal), resources (lead), supervision (lead), writing – original draft (lead), writing – review and editing (equal). **Osvaldo Alexander Gomez‐Gomez:** data curation (equal), formal analysis (equal), investigation (equal), validation (equal), writing – review and editing (equal). **Valentina Montoya‐Jaramillo:** data curation (equal), formal analysis (equal), investigation (equal), methodology (equal), validation (equal), writing – review and editing (equal). **Felipe A. Toro‐Cardona:** data curation (equal), formal analysis (equal), investigation (equal), methodology (equal), writing – review and editing (equal). **Paula Pinzon‐Cardenas:** formal analysis (equal), investigation (equal), methodology (equal), supervision (equal), writing – review and editing (equal).

## Funding

This work was supported by Universidad de Antioquia, Young Researcher Program 2023‐61630.

## Conflicts of Interest

The authors declare no conflicts of interest.

## Supporting information


**Figure S1:** Principal component analysis for morphological variables, indicating along which axis are located.
**Figure S2:** Graphical summary of the 13 models built per response variable. Arrows show interactions between factors.

## Data Availability

The data that support the findings of this study are openly available in Dryad at DOI https://doi.org/10.5061/dryad.g1jwstqtc.
