# Supplementary material for: Disentangling the Evolutionary Cause–Effect Relationships of Environment, Sexual Selection, and Body Size With Birdsong Frequency
Source: Ecol Evol. 2026 Apr 3;16(4):e73351. doi: 10.1002/ece3.73351 (PMC13052002; doi:10.1002/ece3.73351)
Supplement: Supplementary file 1 — Figure S1: Principal component analysis for morphological variables, indicating along which axis are located. Figure S2: Graphical summary of the 13 models built per response variable. Arrows show interactions between factors. [file ECE3-16-e73351-s001.pdf]

Supporting material for: Disentangling the evolutionary cause-effect relationships of environment, sexual selection and body size with birdsong frequency

Hector Fabio Rivera-Gutierrez[1*], Osvaldo Alexander Gomez-Gomez[1], Valentina Montoya-Jaramillo[1], Felipe A. Toro-Cardona[1,2], Paula Pinzon-Cardenas[1]
1 Grupo de Ecología y Evolución de Vertebrados, Instituto de Biología, Facultad de Ciencias Exactas y Naturales, Universidad de Antioquia, Calle 70 Nº 52-21, Medellín, Colombia.
2 Red de Biología Evolutiva, Laboratorio de Bioclimatología, Instituto de Ecología, A.C. Carretera antigua a Coatepec 351, El Haya, Xalapa, 91073, Veracruz, México

*Correspondence: H F Rivera-Gutierrez, fabio.rivera@udea.edu.co

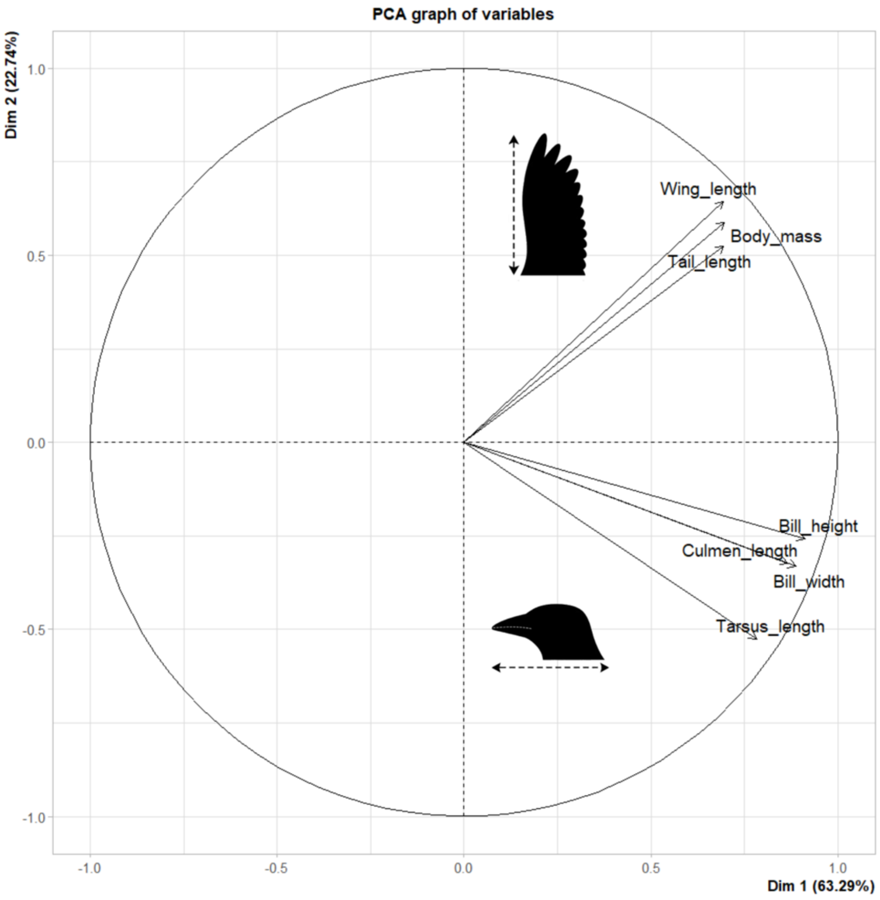

Supporting figure 1. Principal component analysis for morphological variables, indicanting along wich axis are located.

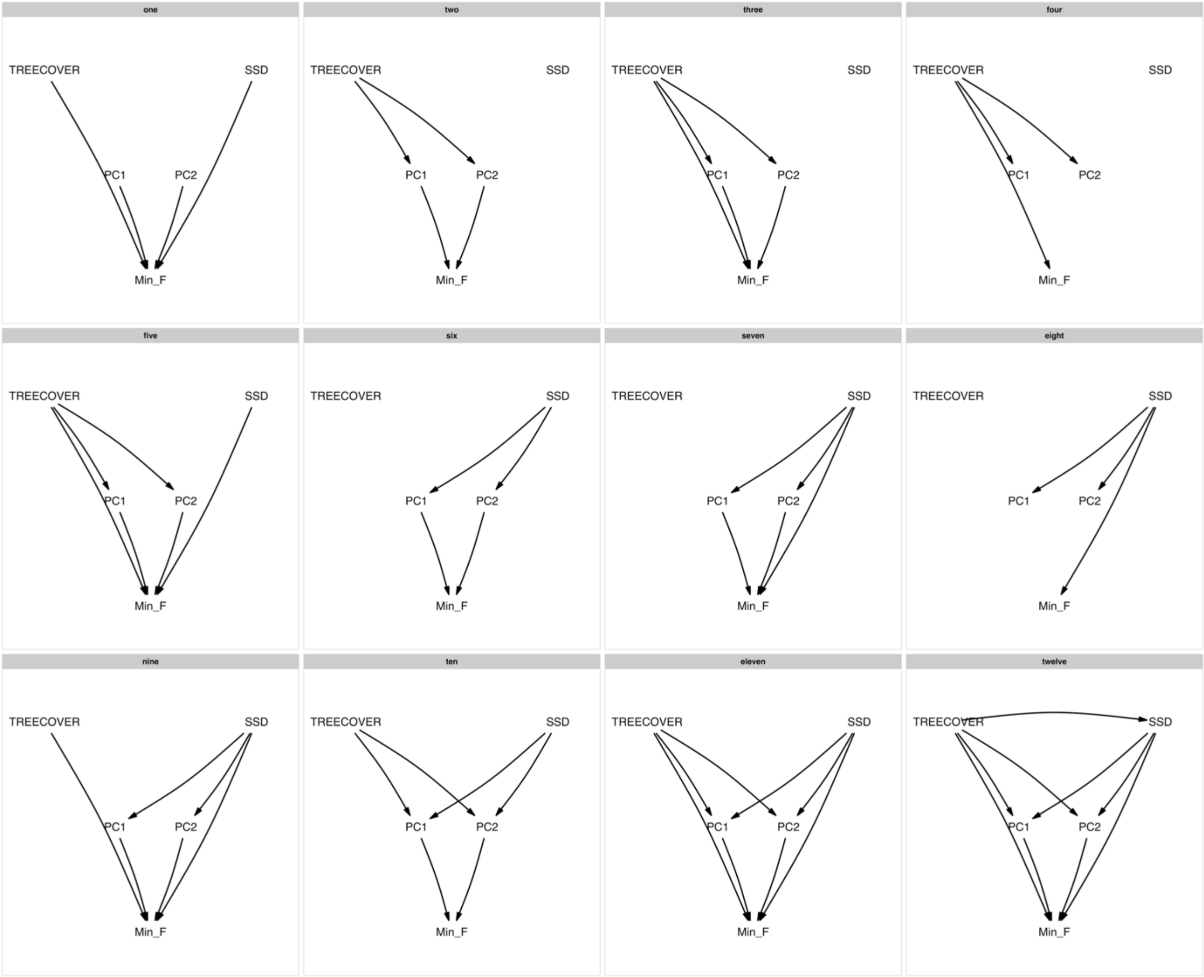

Supporting figure 2. Graphical summary of the 13 models built per response variable. Arrows show interactions between factors.
